# 

**Published:** 1868-10-15

**Authors:** 


Vol. XXV. Number 20

### THE CHICAGO MEDICAL JOURNAL.

*PUBLISHED SEMI-MONTHLY.*

J. Adams Allen, M.D., LL.D.
EDITOR.
Walter Hay, A.M., M.D.
ASSOCIATE EDITOR.

OCTOBER 15, 1868.

CHICAGO:
Church, Goodman and Donnelley, Steam Book and Job Printers,
108 and 110 Dearborn Street.

